# Research on a Wire Rope Breakage Detection Device for High-Speed Operation Based on the Multistage Excitation Principle

**DOI:** 10.3390/s23239298

**Published:** 2023-11-21

**Authors:** Zhou Zhou, Xiuheng Zhang, Ran Deng, Lu Han, Meng Zhou, Zhuangzhuang Ma, Xiangdong Chang, Yuxing Peng

**Affiliations:** 1 Special Equipment Safety Supervision Inspection Institute of Jiangsu Province, Xuzhou 221116, China; joeddong@163.com (Z.Z.); dr0421@163.com (R.D.); brent9597@163.com (L.H.); zmeng92@163.com (M.Z.); 2 School of Mechanical and Electrical Engineering, Jiangsu Key Laboratory of Mine Mechanical and Electrical Equipment, China University of Mining and Technology, Xuzhou 221116, China; zz28zz2810@163.com (Z.M.); changxiangdong@cumt.edu.cn (X.C.); pengyuxing@cumt.edu.cn (Y.P.); 3 Jiangsu Collaborative Innovation Center of Intelligent Mining Equipment, Xuzhou 221116, China

**Keywords:** wire rope, high speed, multistage, excitation, wire break detection

## Abstract

Wire rope breakage, as damage easily produced during the service period of wire rope, is an important factor affecting the safe operation of elevators. Especially in the high-speed elevator operation process, the problem of magnetization unsaturation caused by speed effects can easily lead to deformation of the magnetic flux leakage detection signal, thereby affecting the accuracy and reliability of wire breakage quantitative detection. Therefore, this article focuses on the problem that existing wire rope detection methods cannot perform non-destructive testing on high-speed elevator wire ropes and conducts design and experimental research on a high-speed running wire rope breakage detection device based on the principle of multi-stage excitation. The main research content includes simulation research on the multistage excitation, structural design, and simulation optimization of open–close copper sheet magnetizers and the building of a detection device for wire rope breakage detection experimental research. The simulation and experimental results show that the multistage magnetization method can effectively solve the problem of magnetization unsaturation caused by the velocity effect. The multistage excitation device has a good wire breakage recognition effect for speeds less than or equal to 3 m/s. It can detect magnetic leakage signals with a minimum of four broken wires and has good detection accuracy. It is a new and effective wire breakage detection device for high-speed elevator wire rope, providing important technical support for the safe and reliable operation of high-speed elevators.

## 1. Introduction

As an important component of the elevator traction system, the quality of the wire rope directly affects the safety of elevator operation [1,2,3]. With the rapid development of high-rise buildings, high-speed elevators are widely used. Wire ropes, as load-bearing components, are a direct factor affecting the safe operation of elevators due to wire breakage caused by wear, corrosion, and excessive fatigue during operation [4,5]. According to the wire rope testing and discard criteria, a certain number of broken wires or cross-sectional area losses are unacceptable in engineering applications [6]. The best detection speed for existing wire rope detection instruments (strong magnetic) is 0.3–2 m/s, while the operating speed of high-speed elevators usually exceeds 2.5 m/s. The detection speed of high-speed running wire ropes is far from reaching the operating speed. Therefore, conducting nondestructive testing research on wire ropes during high-speed operation has important practical significance.

The most representative nondestructive testing methods applicable to wire ropes include infrared testing [7], ultrasonic guided wave testing [8], optical testing [9], and electromagnetic testing [10], as shown in Table 1. Among them, electromagnetic testing methods are the most widely used in the field of wire rope nondestructive testing. The existing electromagnetic testing methods include eddy current testing, magnetic memory testing, and magnetic flux leakage (MFL) testing [11,12,13,14]. Magnetic flux leakage testing technology, as an efficient non-destructive testing method, does not require coupling agents. It can not only detect numerous types of defects in the surface, interlayer, and bottom layers of ferromagnetic components but can also determine the geometric shape of defects. At the same time, due to its good stability and anti-interference ability, it does not require high testing environment requirements and can achieve online testing with high automation. Therefore, magnetic flux leakage testing technology is the most suitable electromagnetic testing method for the non-destructive testing of wire ropes [15,16,17]. Zhang et al. designed a three-dimensional MFL acquisition system based on unsaturated magnetic excitation using tunnel magnetoresistive (TMR) components, proposed a three-dimensional MFL detection method, and found that three-dimensional MFL color imaging can effectively improve the recognition rate of wire rope breakages [18]. Kim et al. used envelope processing based on the Hilbert transform to compare the envelope signal with the threshold established based on the generalized extreme value (GEV) distribution, analyze the envelope signal, and quantitatively analyze the detected magnetic flux leakage signals that exceed the threshold [19]. Kaur et al. designed a new type of magnetic flux leakage detection device for wire ropes. Cutting the long axis ring of the Nd-Fe-B permanent magnet into 32 equal arc segments and magnetizing it in parallel in a magnetizer can effectively detect wire rope breakage damage [20]. Yan et al. proposed an electromagnetic nondestructive testing method that simplifies the magnetic circuit, achieving the defect detection of wire ropes under the environment of electromagnetic interference [21]. Sun et al. proposed the C coil open excitation method, which solved the problem of the inconvenient installation of the magnetization device during use [22]. Yan et al. improved the structure of the coil sensor used in traditional magnetic flux leakage detection by adding an internal wedge-shaped iron core, effectively improving the signal-to-noise ratio and the installation convenience of the magnetic flux leakage detection induction coil [23]. Wang et al. proposed a novel magnetic aggregation bridge detection method using magneto resistive (MR) sensor arrays that can detect multiple types of damage caused to wire ropes with a maximum signal-to-noise ratio of 60 dB [24]. Zhang et al. proposed a quantitative calculation method for the fractal dimension of the magnetic leakage field of wire ropes by analyzing the fractal characteristics of the magnetic leakage signal of corroded wire ropes. The research results showed that this technology can accurately determine the degree of corrosion and the circumferential position of wire ropes [25].

Finite element electromagnetic simulation provides theoretical guidance for the actual excitation situation of wire ropes and has been widely applied in scientific research fields. Zhang et al. proposed a new theoretical model of force magnetic coupling based on weak magnetic excitation, which extracts the magnetic signal characteristics of wire rope structures through ANSYS simulation to achieve the quantitative analysis of wire rope damage [26]. Ye et al. calculated the leakage field of weld defects using ANSYS finite element simulation software based on quantitative analysis and discussed the characteristic laws of the defect leakage magnetic field and its components in any magnetization direction [27]. Coramik et al. used ANSYS Maxwell simulation software to examine the effects of crack geometry and magnetization speed on MFL signals and determined the optimal sensor location [28]. Zhou et al. proposed the permanent magnet codirectional excitation weak magnetic method and found that the defect detection sensor based on the permanent magnet codirectional array combining excitation and magnetic focusing functions improved the magnetic signal of defects by 6–8 times through Ansoft simulation [29]. Wang et al. [30] proposes a new method for detecting magnetic excitation in wire ropes by combining the structural models for dynamic magnetic field balancing and magnetic focusing, and the effectiveness of the model was verified through finite element simulation. From the above, scholars’ research on wire rope excitation simulation mostly focuses on static wire rope excitation simulation, studying the internal magnetic field distribution of the wire rope and the distribution of the magnetic field after the excitation of the wire rope by different excitation devices with different structures.

With the increasing height and running speed of elevators, the requirements for magnetic flux leakage detection devices for wire ropes are becoming increasingly high. In magnetic flux leakage testing, when the relative motion speed between the workpiece and the direct-current (DC) magnetizer is greater than 3 m/s, it is considered high-speed magnetic flux leakage testing [31]. The optimal operating speed of existing wire rope flaw detection instruments is mostly within 2 m/s, while the operating speed of high-speed elevators is usually within 3 m/s. Existing wire rope damage instruments cannot meet the condition detection of high-speed elevator wire ropes [32]. Therefore, it is urgent to develop a set of non-destructive testing equipment for high-speed elevator traction wire ropes, providing important technical support for the safe and reliable operation of elevators. Ji et al. [33] proposed an improved adaptive filtering method that reduces the interference caused by vibration during high-speed detection by more than 80%. Liu et al. [34] improved the sensor head using an open magnetizer, combining magnetic sensing coupling with weak analog signal processing, and successfully applied it to the high-speed detection of wire ropes. Tian et al. [35] optimized the excitation model parameters of the sensor using the axisymmetric finite element analysis method, increasing the detection speed to 5 m/s. However, the phenomenon of magnetization unsaturation caused by the velocity effect has not been resolved. Existing research mostly focuses on the non-destructive testing of mine hoist steel wire ropes, but there is currently little research on the non-destructive testing of steel wire ropes based on the actual working conditions of high-speed traction elevators.

In response to the phenomenon of magnetization unsaturation caused by the speed effect in high-speed running wire ropes, this paper conducts design and experimental research on a high-speed running wire rope breakage detection device based on the principle of multistage excitation. The main research content includes simulation research on the multistage excitation, structural design, and simulation optimization of an open–close copper sheet magneto and the set-up of the detection device for wire breakage detection experimental research. The research results can provide feasible solutions for the saturation excitation and rope breakage signal detection of wire ropes in high-speed operation. This research has important data support and theoretical significance for evaluating the use status of wire ropes.

## 2. Simulation Study of Wire Rope Excitation

### 2.1. Wire Rope Excitation Simulation

The traditional method for detecting wire rope damage usually uses single-stage excitation technology, which applies an excitation signal to the entire wire rope and then obtains the damage information of the steel wire rope by detecting the echo signal, but is unable to solve problems such as complex electromagnetic phenomena caused by speed increases and signal distortion caused by dynamic magnetization. The multi-stage excitation technology adopts the method of multiple excitation signals, which improve the accuracy and reliability of detection by applying multiple excitation signals at different positions. To compare and analyze the excitation characteristics of steel wire ropes under single stage and multi-stage electromagnets, a finite element model of steel wire rope excitation simulation was established based on Creo 8.0 3D drawing software and Ansys Maxwell electromagnetic simulation software (v. 2023).

**Figure 1:**
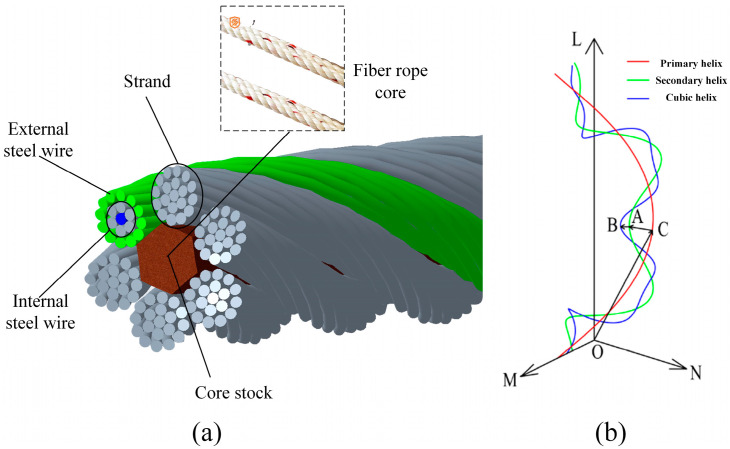
Structure of the test wire rope and the wire rope space helix: (**a**) the structure of the wire rope, (**b**) the required spatial three spiral curves for the wire rope.

The wire rope used in the experiment is 6 × 19 + FC hot-dip galvanized hemp core wire rope, which is twisted in a right alternating manner with a diameter of 9.3 mm, a steel wire diameter of 0.6 mm, and a lay distance of 70 mm. The main components of steel wire materials include 94% Fe, 4.53% Zn, 0.87% C, 0.39% Mn, 0.02% Si, 0.01% Ni, and a very small amount of S and P. The whole rope is made of six strands of rope and one strand of cotton hemp material twisted into a rope core, and each strand is made of 19 steel wires twisted through a special process. The structure of the wire rope is shown in Figure 1a. The Frenet standard frame and Frenet equation set are used to establish the parametric equations of the three spiral curves, and the parametric equations of the computational formula are used to establish the wire rope model. The required spatial three spiral curves for the wire rope are shown in Figure 1b.

**Figure 2:**
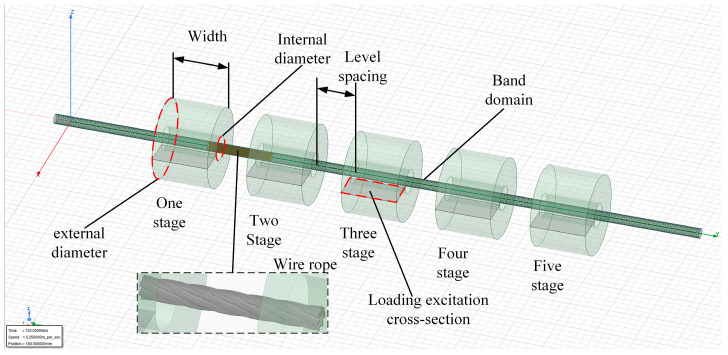
Simulation model of the multistage excitation of wire rope.

The drawn 3D model of the wire rope is imported into ANSYS Maxwell, and a transient solver is set up. To reduce the simulation time and optimize the simulation process, the 3D model of the coil is simplified into a solid cylindrical structure. The steps include the following: Draw a 5-stage electromagnet and band domain, define the motion parameters as linear motion, and set the outer diameter of the electromagnet to 30 mm, the width to 70 mm, and the spacing between each stage to 50 mm. Divide the excitation loading cross-section and set the excitation type as a constant DC source, with a parameter of 2 A and 1000 turns, flowing downward from the vertical cross-section. The material properties of the wire rope and electromagnet are set separately, and the software’s adaptive mesh generation function is applied to separate the mesh of the wire rope and electromagnet. Finally, the solution domain, solution step, and save step are set for simulation, and the eddy current field cloud map operation is generated through postprocessing. The multistage excitation simulation model of the wire rope is shown in Figure 2.

### 2.2. Simulation Analysis of the Transient Eddy Current Field under Different Speeds and Magnetic Stages

**Figure 3:**
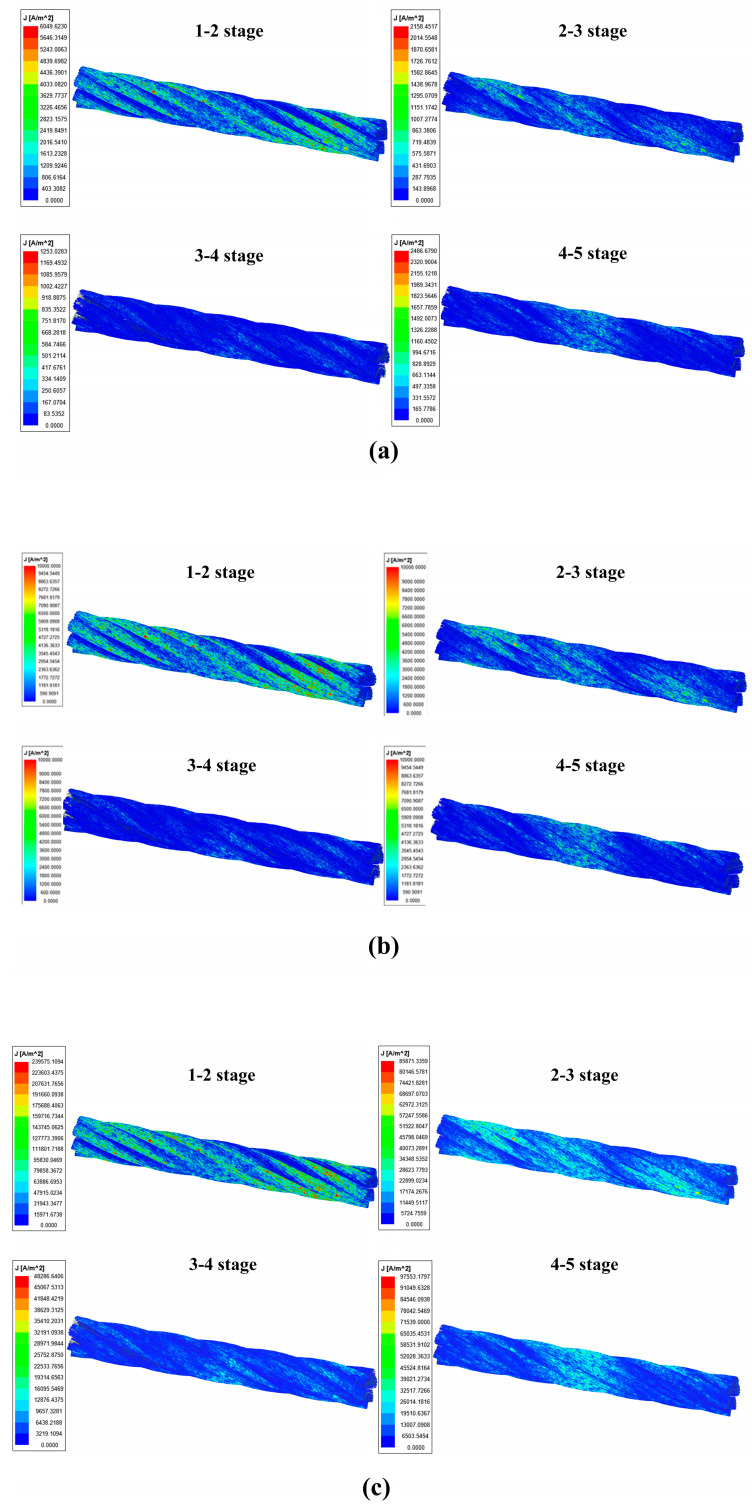
Cloud diagram of the vortex field in classes 1–5 at 0.25 m/s, 3 m/s, and 10 m/s: (**a**) 0.25 m/s, (**b**) 3 m/s, (**c**) 10 m/s.

The motion parameters are set to 0.25 m/s (low speed), 3 m/s (high speed), and 10 m/s (ultrahigh speed) for the excitation simulation, and the cloud diagrams of the wire rope kinetic eddy current field under different speeds and magnetization stages are obtained, as shown in Figure 3. It was found that under low, high, and ultrahigh operating speeds, the distribution of the induced eddy current field generated by the relative motion of the wire rope and the magnetizer is basically consistent between each stage.

Based on the eddy currents in wire ropes at different speeds between stages 1 and 2, the maximum values of the eddy current field at low, high, and ultrahigh speeds are 6095 A/m^2^, 10,000 A/m^2^, and 239,875 A/m^2^, respectively. According to the theory of the velocity effect, the electrons in the wire rope are subjected to the Lorentz force of the magnetic field and generate eddy currents spontaneously, and at this time, the internal current density $\hat{J}$ The formula can be expressed as

(1) $$\hat{J}=\hat{J_{s}}+\hat{J_{e}},$$

where $\hat{{\;J}_{s}\;}$ is the source current generated by the external magnetic field ($A/m^{2}$). $\hat{J_{e}}$ is the eddy current generated by the relative motion of the magnetizing unit and the wire rope under test ($A/m^{2}$).

Combined with Faraday’s law of electromagnetic induction, we can derive

(2) $$\nabla \times E=\nabla \times (\hat{E}+v\times \hat{B})=-\left(\frac{\partial \hat{B}}{\partial t}+(v\cdot \nabla )\hat{B}\right),$$

Combined with the electric field strength $\overset{`}{E}=-\frac{\partial \overset{`}{A}}{\partial \overset{`}{t}}$ formula, it is easy to derive

(3) $$\nabla \times H={\hat{J}}_{s}+\sigma \left(-\frac{\partial \hat{A}}{\partial \bar{t}}+v\times \hat{B}\right),$$

Combined with $\frac{1}{\mu }\nabla^{2}\cdot A=-J$, the eddy current dynamic differential equation in the reference system of the magnetized coil at a constant magnetic field is obtained as follows:

(4) $$\frac{1}{\mu }\nabla^{2}\cdot A=-{\hat{J}}_{S}-\sigma v\times \nabla \times \hat{A},$$

By combining the formula and simulation results, it can be concluded that when using the coil magnetization component as the reference frame, the magnitude of the eddy current is related to *v* × ∇ × *A*. With increasing speed, the intensity of the eddy current field increases. However, compared to low speed, the increase in the eddy current field of the wire rope under high-speed operation is greater.

Based on an analysis of the cloud diagram of the eddy current field at 10 m/s between stages 2 and 5, it can be seen that the maximum value of the eddy current field between stages 1 and 2 is 239,875 A/m^2^, and that between stages 2 and 3 is 85,871 A/m^2^. According to the theory of multistage excitation, the eddy current generated during the axial movement of the wire rope is

(5) $$J=\frac{\varepsilon }{R}=-\frac{v_{z}}{2\pi \rho r}\iint_{s}\;\frac{dB_{z}}{dz}ds,$$

where $v_{z}$ is the axial component of the velocity and $B_{z}$ is the axial component of the magnetic field of the coil.

Therefore, the magnitude of eddy currents in the wire rope is related to the rate of change of the external magnetic field. The existence of eddy currents affects the magnetization field of the coil on the wire rope at different positions. The eddy current field is larger at the edge of the coil and smaller at the center of the coil. Based on the vector superposition of the coil magnetic field, the rate of change of the magnetic field decreases, reducing the intensity of the eddy current field generated in the wire rope. Under the conditions of this simulation test, the maximum reduction in eddy current is achieved at stages 2 and 3. It can be concluded that multistage magnetization can reduce the generation of eddy currents and increase the magnetization effect of wire ropes. Therefore, the use of multistage magnetization devices can enhance the ability of wire rope to detect wire rope breakages.

## 3. Structural Design and Excitation Simulation of a Multistage Excitation Device

### 3.1. Multistage Excitation Device Structure Design

In the magnetic flux leakage nondestructive testing of wire ropes, due to the different diameters of the wire ropes, there are drawbacks such as uneven magnetization and excessive magnetic attraction in the magnetic circuit design process using permanent magnets as magnetization sources. Although the direct current type passing through the coil can solve the problems of uneven magnetization and high suction, the closed structure of the coil cannot pass through the circular wire rope, making online detection implementation difficult. Therefore, it is necessary to optimize the DC coil based on its poor adaptability to detection conditions.

**Figure 4:**
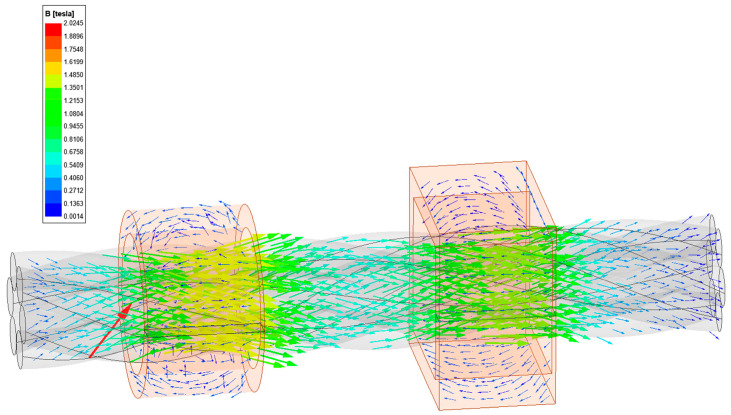
Magnetic field cloud of C-type and rectangular cross-section coils.

To ensure that the width, thickness, and inner diameter of the coil remain unchanged, C-shaped and rectangular cross-section coils are used to carry out the excitation simulation study on the wire rope. Figure 4 shows the simulation cloud map of the magnetic field of C-shaped and rectangular cross-section coils. As shown in the figure, the C-shaped coil has a stronger magnetic field and a better magnetization effect on the wire rope, so its shape is optimized to be C-shaped. At the same time, based on the closed-loop structure of the wire rope, the coil needs to pass through the wire rope and adapt to different lay lengths, so the coil magnetizer should be optimized as a width-adjustable structure.

**Figure 5:**
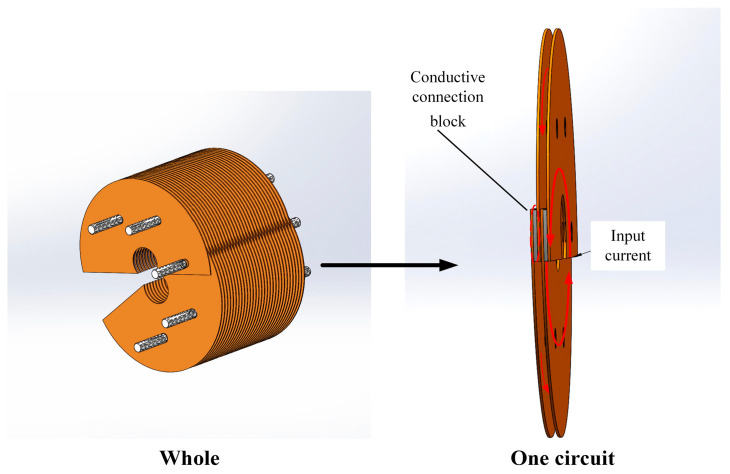
Schematic diagram of the structure of the open and closed copper sheet magnetizer.

Figure 5 is a schematic diagram of the final optimized open–close copper sheet magnetizer structure, which is composed of a solenoid-like structure consisting of multiple ring coil circuits. It can open and close freely and is fixed by tightening insulation bolts and nuts to achieve current flow. To reduce the swing of the wire rope, the magnetizer is generally placed near the crown wheel or guide wheel, but the vibration amplitude of the high-speed running wire rope is 10 mm up and down, so the inner diameter of the magnetizer is determined to be 30 mm. The open–close copper plate-type magnetizer consists of multiple circuits; each circuit can be added or subtracted, and the number of copper plates can be adjusted under actual conditions.

### 3.2. Excitation Simulation

**Figure 6:**
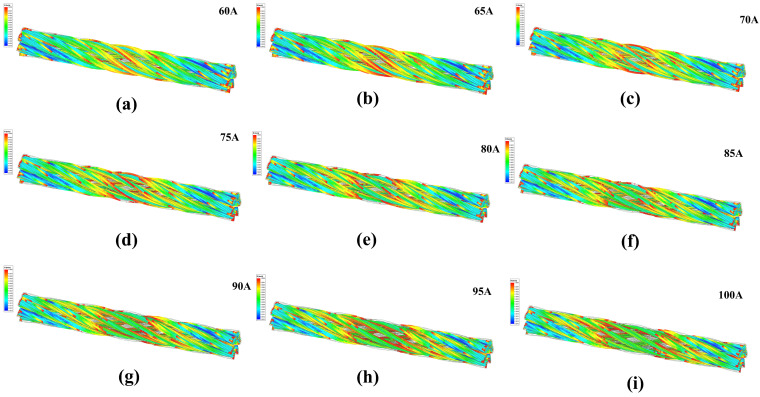
Magnetic field cloud in the wire rope under different current strengths: (**a**) 60 A, (**b**) 65 A, (**c**) 70 A, (**d**) 75 A, (**e**) 80 A, (**f**) 85 A, (**g**) 90 A, (**h**) 95 A, (**i**) 100 A.

An excitation simulation was performed on the wire rope based on the outer diameter and current parameters. The operating speed of the wire rope was 8 m/s, with a magnetic field between stages 2 and 3, a current range of 60–100 A, a step size of 5 A, and a magnetic field threshold of 1 T. The magnetic induction intensity magnetic field scalar cloud map in the middle range of the wire rope is shown in Figure 6.

Comparing the magnetic induction cloud map of the wire rope at 60–100 A currents, it can be seen that with increasing current (60–75 A), the excitation effect is obvious. After the current increases to 75 A, the excitation slows down, which is similar to the magnetization curve of ferromagnets. This is because after the current reaches 75 A, as the current increases, eddy currents appear compared to the magnetic field strength, hindering the establishment of a saturated magnetic field and weakening the excitation intensity in the middle section.

**Figure 7:**
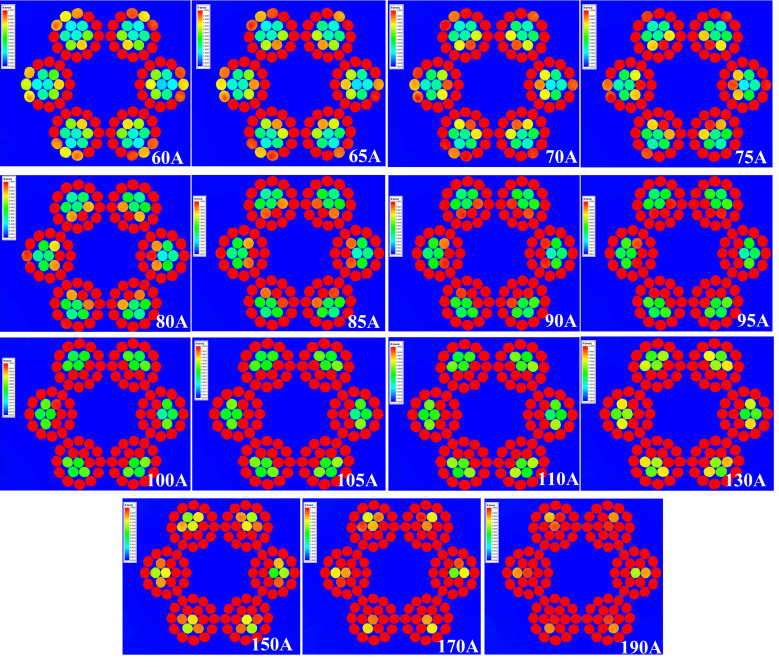
Magnetic field cloud in the cross-section of wire rope under different current strengths.

To study the distribution and changes of the internal magnetic field of the wire rope, the cross-section of the wire rope was cut open, as shown in Figure 7. Under a current of 60–170 A, the magnetic field diffuses outward from the axis of the wire rope in the radial direction, and the internal steel wire saturates first. Due to the spatial spiral structure of the wire rope, the internal steel wire and the outer wire rope breakage are the same spatial body, and the magnetic force line flows to the outer layer. The current is approximately 75 A, and the magnetic field of the outermost steel wire reaches saturation; when it reaches 190 A, the wire rope as a whole tends to saturate. To study the quantitative detection of external wire breakage, only the magnetic saturation of the external steel wire is needed. Therefore, a current of 75 A is selected for the wire breakage detection test.

**Figure 8:**
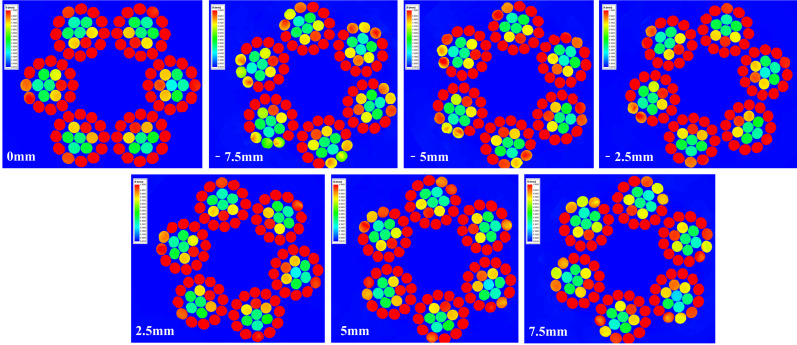
Magnetic field clouds in different sections of wire rope at 75 A current strength.

To thoroughly analyze the range of the magnetic field distribution in the wire rope under 75 A current, divide the magnetic field domain of the wire rope from the midpoint and obtain magnetic field cloud maps of the wire rope cross-section at different positions, as shown in Figure 8. It was found that the magnetic field of the outermost steel wire exceeded 1 T and reached a width of over 10 mm, while the width of the test interrupted wire was often smaller than the magnetic field domain. Therefore, under a current of 75 A, the excitation saturation field range of the magnetizer met the requirements for wire breakage detection.

**Figure 9:**
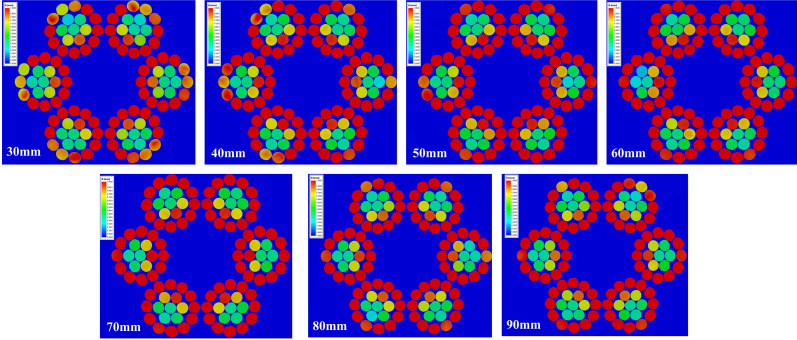
Magnetic field cloud of the wire rope cross-section for different outer diameters under 75 A current.

To meet the requirement that the cross-section of the copper sheet can pass the 75 A current parameter, the initial value of the outer radius is determined to be 30 mm. The fixed current parameter of 75 A remains unchanged, and the external radius is parameterized for simulation (30–100 mm step 10 mm), as shown in Figure 9. The magnetic field distribution of the wire rope cross-section shows that as the outer radius increases, the magnetic induction intensity in the wire rope increases. The outer diameter of the excitation and the magnetic field strength are positively correlated, and the magnetic field is saturated when the outer diameter is 50 mm. Therefore, it is determined that the outer radius of the open–close copper magnetizer is 50 mm.

## 4. Experimental Study on Broken Wire Detection

### 4.1. Interrupted Filament Detection Test for Different Magnetic Grades

**Figure 10:**
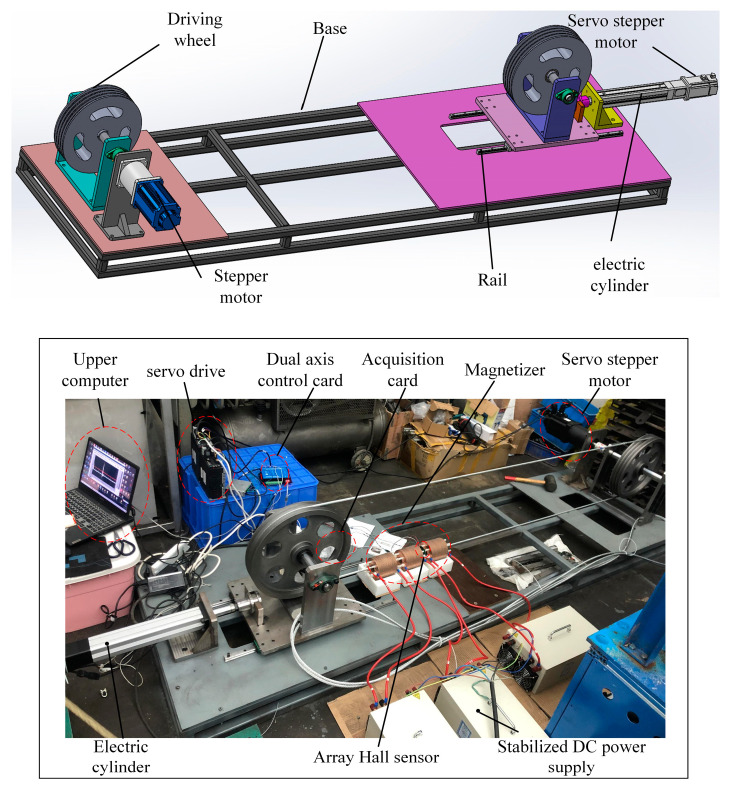
High-speed running wire rope inspection test bench.

Based on the simulation results, a nondestructive testing device for high-speed running wire ropes is constructed for wire breakage detection testing. The overall structure of the high-speed running wire rope test bench is shown in Figure 10. The transmission mode adopts an open-type transmission. To achieve strong magnetic field requirements and reduce interference, three regulated DC adjustable power supplies are installed to excite the magnetizer to generate a strong magnetic field. The magnetized wire rope generates a leakage magnetic field at the wire rope breakage. The leakage magnetic signal collected by the Hall element is converted into voltage and transmitted to the acquisition card through an external circuit. It is converted into a digital signal through A/D conversion in the acquisition card and displayed on the upper computer interface.

**Figure 11:**
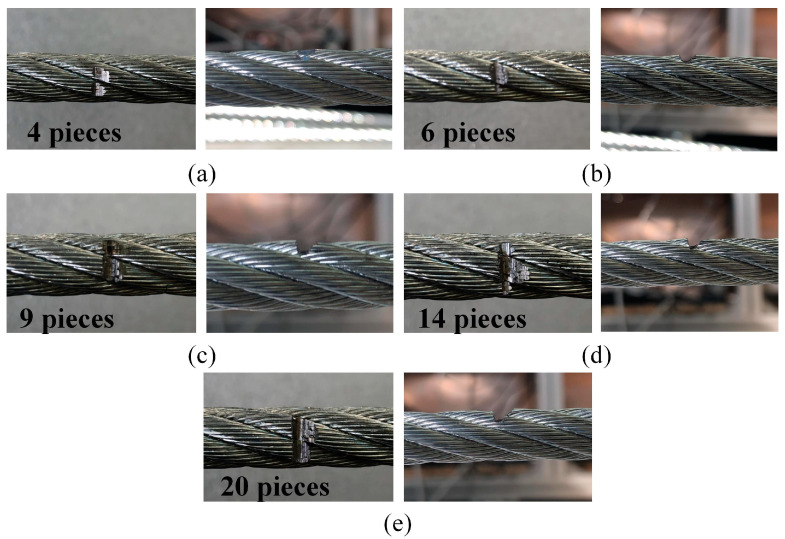
Wire rope breakage specimen: (**a**) 4 pieces, (**b**) 6 pieces, (**c**) 9 pieces, (**d**) 14 pieces, (**e**) 20 pieces.

The acquisition card captures the signal of the wire rope moving between the two rollers for one cycle. The range of the acquisition card is set to 5 V, and the sampling rate is 2000 sps/s. Based on the collected signals, the number of wire rope breakages is identified, and Hall elements are used to detect abnormal points in the wire rope signal. The characteristics of the signal abnormal points are compared with the characteristics of typical wire rope breakage signals, and numerical calculations are performed to determine the number of wire rope breakages. The outer layer of the wire rope was cut to prepare 4 (a), 6 (b), 9 (c), 14 (d), and 20 (e) broken wire samples, as shown in Figure 11.

**Figure 12:**
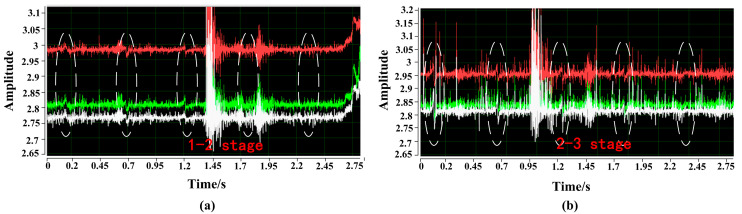
Raw signal of magnetic leakage between different stages of wire rope breakage at a speed of 1.36 m/s: (**a**) stages 1–2, (**b**) stages 2–3.

Figure 12 shows the original signal of the magnetic flux leakage between different stages of wire rope breakage at a speed of 1.36 m/s. The horizontal axis represents the detection time (s), and the vertical axis represents the voltage (v) of the Hall sensor. From the original signal graph, it can be seen that the signal is composed of noise and useful magnetic flux leakage signals. The detection device detects different wire rope breakage magnetic flux leakage signals under a sudden change similar to a sine wave signal. Due to the axial excitation of the wire rope by the magnetizer, the magnetic field lines leak from the wire rope breakage gap, and the placement of the three Hall elements makes the sensitive direction perpendicular to the magnetic field lines. The magnetic field line passes through the allergic surface and returns to the wire rope through external air, resulting in a sudden signal with two peaks.

Based on a comparison of the magnetic leakage signals of different wire rope breakages detected between two different magnetic stages, it was found that stages 1 and 2, more than nine wire rope breakage detections were obvious, while the signals of four and six wire rope breakages were not obvious and were even submerged in noise. The magnetic flux leakage signal of four wire rope breakages were accurately detected between stages 2–3, and the signal-to-noise ratio was high. It can be seen that multistage excitation makes the magnetic field in the wire rope more saturated, resulting in more magnetic flux leakage. The results are consistent with the simulation.

**Figure 13:**
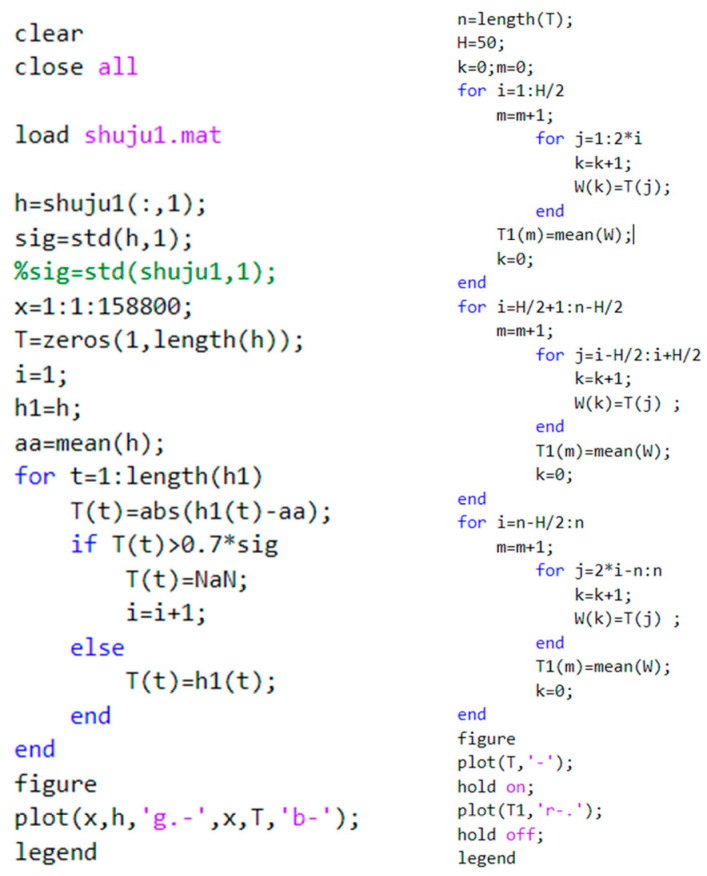
Moving window mean filtering algorithm.

**Figure 14:**
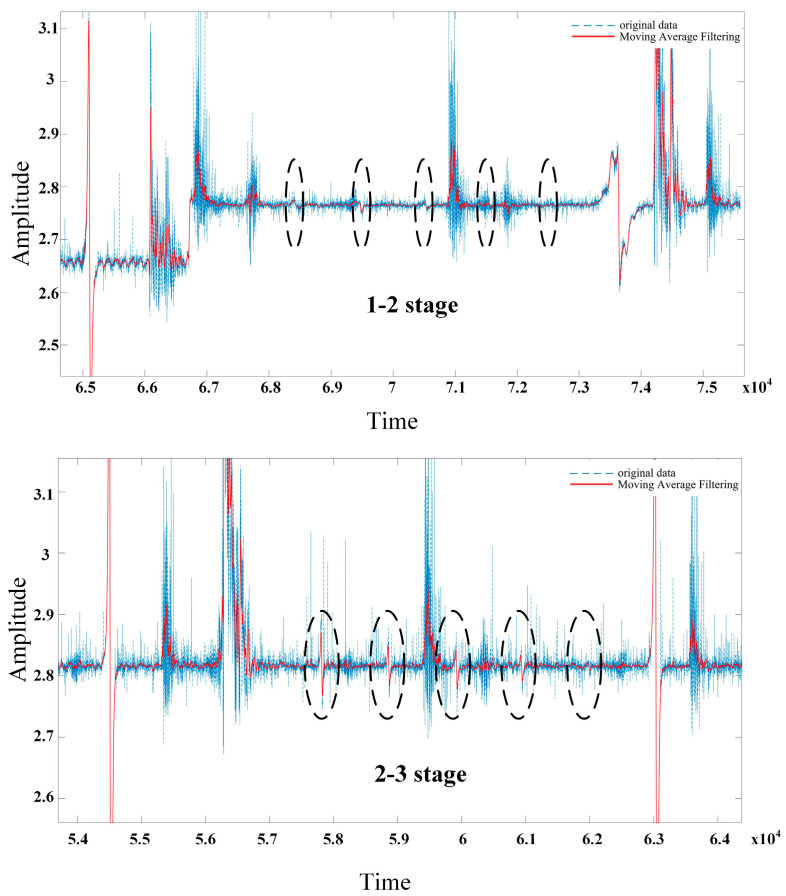
Magnetic leakage signal after noise reduction and filtering.

When defective steel wire rope is saturated with magnetization, the surface generated leakage magnetic field information is more complex, including the leakage magnetic field caused by broken wires, external background interference, etc. Some of these leakage magnetic field signals are seriously affected by noise compared to the broken wire leakage magnetic field signals. Therefore, wavelet transformation and moving window mean filtering are used to process the noise and obtain useful signals, as shown in Figure 13. The extraction of feature signals is achieved through Labview. The first step is to read the data from the acquisition card and intercept a length signal. Next, based on the amplitude and level controls, an appropriate threshold V is set in combination with the magnetic flux leakage signal. This threshold is used to shield the interference of small peaks in the surrounding signal, identify the positive and negative peaks of the signal, and extract the peaks and valleys in the signal. Finally, the peak-to-peak values of the magnetic flux leakage signals of different stages of interrupted wires are obtained. As shown in Table 2, the peak-to-peak values of detecting different wire rope breakage signals between stages 2 and 3 are 3–6 times the relationship between stages 1 and 2, further proving that multistage excitation is more conducive to signal detection. Figure 14 shows the magnetic flux leakage signal after denoising and filtering. It can be seen that wavelet denoising combined with moving window mean filtering can effectively remove burrs, reduce high-amplitude sudden noise and smooth strand noise, retain the amplitude of the sudden signal of the wire rope breakage, and facilitate the extraction of signal features. However, the reduction of random noise caused by circuit manufacturing problems is not very effective. Due to the fact that random noise signals exhibit a wider signal time domain than broken wire magnetic flux leakage signals and that the waveform does not have symmetrical positive or negative peaks, the impact on magnetic flux leakage signals is relatively small and does not affect the recognition of feature extraction.

### 4.2. Wire Breakage Detection Test at Different Operating Speeds

**Figure 15:**
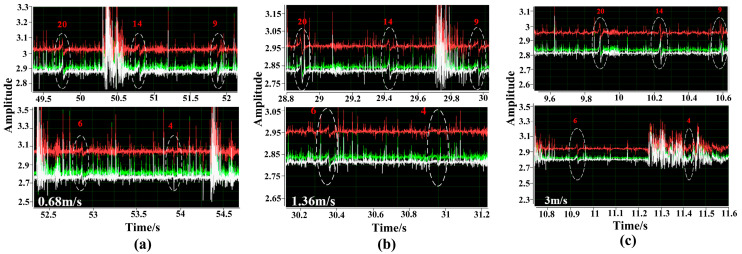
Raw signal of wire rope breakage and magnetic leakage at a speed of 0.68 m/s, 1.36 m/s and 3 m/s: (**a**) 0.68 m/s, (**b**) 1.36 m/s, (**c**) 3 m/s.

**Figure 16:**
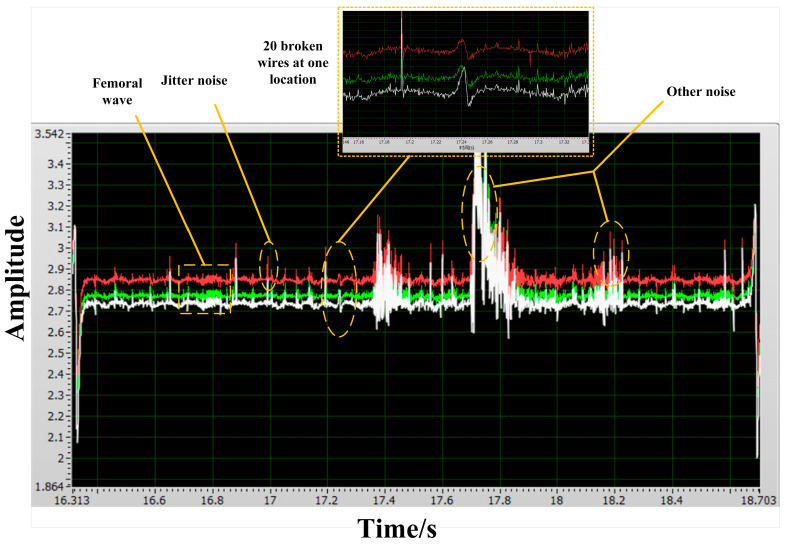
Raw signal of wire rope breakage and magnetic leakage at a speed of 4.2 m/s.

The Hall array signal acquisition circuit was fixed to the multistage magnetizer between stages 2 and 3 to obtain the magnetic flux leakage signals of wire rope breakages between stages 2 and 3 at speeds of 0.68 m/s, 1.36 m/s, 3 m/s, and 4.2 m/s. Figure 15a–c show the wire rope breakage magnetic flux leakage signals of the circular wire rope intercepted at speeds of 0.68 m/s, 1.36 m/s, and 3 m/s for one cycle of operation. Figure 16 shows the original signal of the wire rope breakage magnetic flux leakage at a speed of 4.2 m/s.

By comparing the results of different wire breakage detections at different speeds, it can be concluded that the wire breakage detection device designed in this paper based on the principle of multistage excitation has good detection ability for speeds up to 3 m/s, and four wire rope breakages can be well identified. When the speed is higher than 3 m/s, the ability to detect wire rope breakages is weak. The wire rope breakage signals are submerged in the wave and vibration noise, making it difficult to extract them. Only 20 wire rope breakage leakage signals are detected. This is because the swinging noise caused by the tensile deformation of the wire rope as a flexible component and the impact of the buckle rolling over the roller result in a poor detection ability.

**Figure 17:**
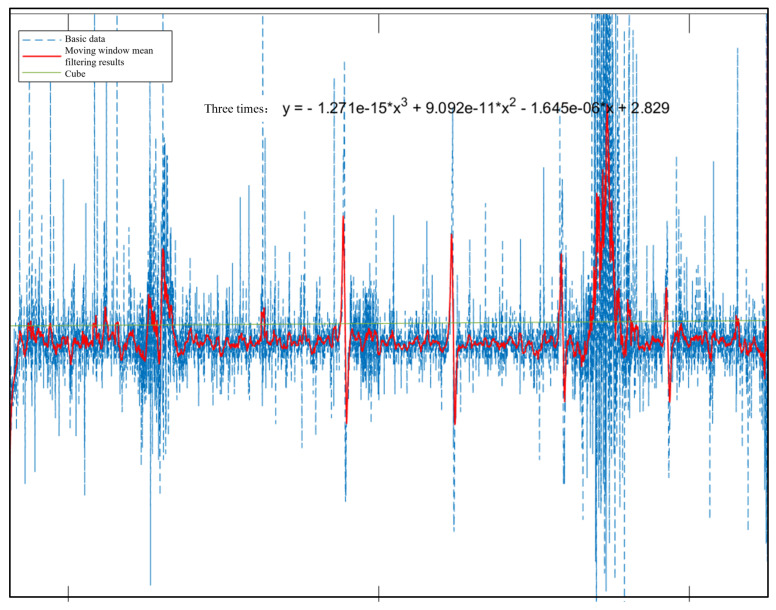
Signal after sliding window mean filtering.

In magnetic flux leakage testing, when the relative motion speed between the workpiece and the DC magnetizer reaches 3 m/s, it is considered high-speed magnetic flux leakage testing. The quantitative analysis of magnetic leakage signals from wire rope breakage at a speed of 3 m/s was carried out by fitting curves. Figure 17 shows the fitted curve of the wire rope breakage leakage signal at a speed of 3 m/s after the combination of wavelet noise reduction and sliding window mean filter denoising. Due to the complex morphology and special spatial structure of wire ropes, which are different from steel rods and steel pipes, there is no linear relationship between the characteristics in the damage signal and the number of wire rope breakages. The peak-to-peak value of the wire rope breakage leakage signal is shown in Table 3.

**Figure 18:**
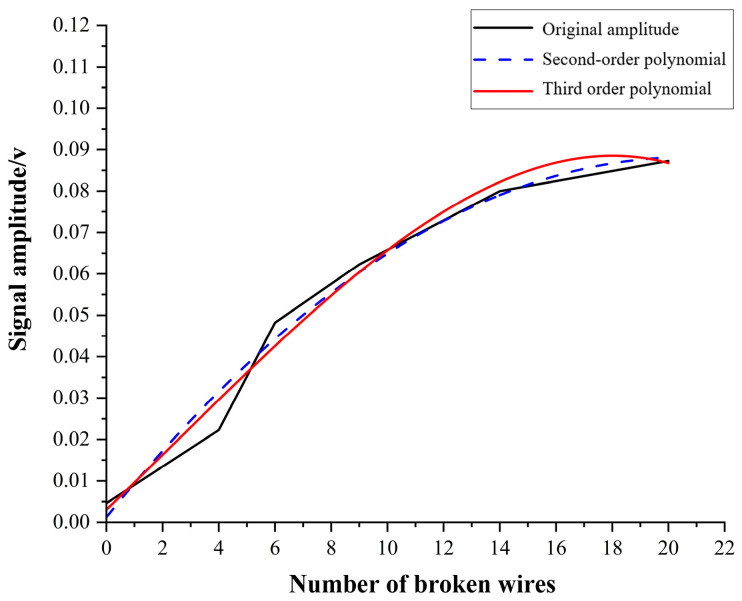
Variation in the signal peak-to-peak value with the number of wire rope breakages.

The polynomial fitting method was used to fit the number of wire rope breakages and the peak-to-peak value of the signal. The relationship between the peak-to-peak value of the signal and the change in the number of wire rope fractures was obtained, as shown in Figure 18. It was found that the coefficient of determination (R^2^) of the curve fitted using a third-order polynomial is 0.98165 by analyzing the three curves in the graph, which is higher than the value (0.97748) of the second-order polynomial. Therefore, the result of third-order polynomial fitting is chosen as the relationship between the number of wire rope breakages and the peak-to-peak value of the signal, that is, Equation (6) is a quantitative identification function. The third-order polynomial fitting function relationship is as follows:

(6) $$f_{2}\left(x\right)=0.00305+0.00652x+6.65054\times 10^{-5}x^{2}-9.15524\times 10^{-6}x^{3}$$

The curve drawn by this quantitative recognition function shows a monotonically increasing trend, which is consistent with theoretical analysis. There are many factors that affect the magnetic flux leakage signal, such as the excitation intensity, noise interference, and lift-off value, among which the lift-off value has the greatest impact. Moreover, due to the high detection speed, the fitting function is only applicable to the nondestructive testing device designed in this article.

## 5. Conclusions

This article applies the principle of magnetic flux leakage testing in non-destructive testing to detect the fracture of high-speed elevator steel wire ropes. Based on the principle of multi-level excitation, a new type of multi-level magnetization device was designed and the optimal structural parameters were determined through simulation. Based on the actual working conditions of high-speed traction elevators, a high-speed running steel wire rope test bench was designed to conduct research on wire rope fracture detection. The effectiveness of the new multi-stage magnetization device designed in this paper for wire rope fracture detection was verified, effectively solving the problem of magnetization unsaturation caused by the speed effect. This provides a feasible solution for the saturation excitation and wire breakage signal detection of steel wire ropes during high-speed elevator operation. The simulation results indicate that the size of eddy currents in the wire rope is related to the relative motion speed of the magnetizer and the rate of change of the external magnetic field. The higher the speed, the stronger the eddy current field. The multistage excitation method can reduce the rate of magnetic field change between the two stages, reduce the generation of eddy currents, and increase the intensity of the excitation magnetic field through the superposition of magnetic field vectors. The superimposed magnetic field generated by the open–close copper magnetization device designed in this article spreads from the middle along the diameter to the surrounding area. With increasing current (60–75 A), the excitation effect is obvious. After the current reaches 75 A, eddy currents hinder the establishment of a saturated magnetic field, weakening the excitation intensity in the middle section. When the current is 190 A, the overall trend of the wire rope is toward magnetic saturation.

The experimental results show that multi-stage excitation effectively improves the accuracy of damage signal recognition. At a speed of 1.36 m/s, only nine or more wire rope fractures can be identified between stages 1 and 2. However, the magnetic flux leakage signal of four broken steel wire ropes can be accurately detected between stages 2 and 3, with a signal amplitude of 3–6 times that of stages 1 and 2. When conducting wire breakage detection on steel wire ropes at different operating speeds, it was found that the device has good recognition ability and detection accuracy for wire rope breakage at speeds below 3 m/s and can effectively detect four broken wires. Therefore, its optimal detection speed is within 3 m/s, making it an effective new type of high-speed elevator wire rope breakage detection device.

## Figures and Tables

**Table 1:** Nondestructive testing methods.

Method		Operating Principle	Advantage	Disadvantage
Infrared detection method		Extracting Infrared Feature Parameters of Objects	No need for contact measurement	High cost
Acoustic emission method		Measurement of ultrasonic waves emitted by structural changes in objects		Only valid in the static load section
Current method		Measuring the Ohmic Resistance of Objects	Measurement of the cross-sectional condition	Difficulty in detection
Ultrasonic guided wave testing		Ultrasonic wave propagation in medium		Unable to reflect the overall situation
Optical detection		Camera detection	High detection accuracy	High cost
X-ray method		Strong X- or γ-ray vertical radiation object	Detect broken wires	High cost and inability to continuously measure
Electromagnetic testing method	Eddy current testing method	Eddy current effect	No need for contact measurement	Only surface damage detected
	Magnetic memory detection method	Magnetic memory effect	No external incentives required	Susceptible to external magnetic field interference
	Magnetic flux leakage testing method	Hysteresis phenomenon	High detection accuracy	Large device volume

**Table 2:** Peak-to-peak values of magnetic leakage signals for different stages of interrupted filaments.

Number of Broken Wires (Pieces)	Peak-to-Peak Value of Signal between Stages 1 and 2 (V)	Peak-to-Peak Value of Signal between Stages 2 and 3 (V)
4	0.00437	0.01309
6	0.00821	0.03917
9	0.01369	0.06205
14	0.0169	0.06643
20	0.0175	0.09952

**Table 3:** Peak-to-peak values of the wire rope breakage damage signals.

Number of Broken Wires (Pieces)	Peak-to-Peak Value of the Signal between Stages 1 and 2 (V)
0	0.00463
4	0.02226
6	0.04821
9	0.06226
14	0.08
20	0.08726

## Data Availability

Data are contained within the article.

## References

[1] Tian J., Zhao C., Wang W., Sun G. (2021). Detection Technology of Mine Wire Rope Based on Radial Magnetic Vector with Flexible Printed Circuit. IEEE Trans. Instrum. Meas..

[2] Peng Y., Huang K., Ma C., Zhu Z., Chang X., Lu H., Zhang Q., Xu C. (2023). Friction and Wear of Multiple Steel Wires in a Wire Rope. Friction.

[3] Peterka P., Krešák J., Vojtko M. (2018). Experience of the Crane Steel Wire Ropes Non-Destructive Tests. Adv. Sci. Technol. Res. J..

[4] Zhang D., Zhang E., Yan X. (2021). Quantitative Method for Detecting Internal and Surface Defects in Wire Rope. NDT E Int..

[5] Zhou P., Zhou G., Zhu Z., He Z., Ding X., Tang C. (2019). A Review of Non-Destructive Damage Detection Methods for Steel Wire Ropes. Appl. Sci..

[6] Liu S., Chen M. (2023). Wire Rope Defect Recognition Method Based on MFL Signal Analysis and 1D-CNNs. Sensors.

[7] Lu S., Zhang J. (2019). Quantitative Nondestructive Testing of Wire Ropes Based on Features Fusion of Magnetic Image and Infrared Image. Shock. Vib..

[8] Rostami J., Tse P.W., Yuan M. (2020). Detection of Broken Wires in Elevator Wire Ropes with Ultrasonic Guided Waves and Tone-Burst Wavelet. Struct. Health Monit..

[9] Zhou P., Zhou G., He Z., Tang C., Zhu Z., Li W. (2019). A Novel Texture-Based Damage Detection Method for Wire Ropes. Meas. J. Int. Meas. Confed..

[10] Zhang Y., Jing L., Xu W., Zhan W., Tan J. (2019). A Sensor for Broken Wire Detection of Steel Wire Ropes Based on the Magnetic Concentrating Principle. Sensors.

[11] Liu S., Sun Y., Jiang X., Kang Y. (2020). A Review of Wire Rope Detection Methods, Sensors and Signal Processing Techniques. J. Nondestruct. Eval..

[12] Feng J., Li F., Lu S., Liu J., Ma D. (2017). Injurious or Noninjurious Defect Identification from MFL Images in Pipeline Inspection Using Convolutional Neural Network. IEEE Trans. Instrum. Meas..

[13] Ni Y., Zhang Q., Xin R. (2020). Magnetic Flux Detection and Identification of Bridge Cable Metal Area Loss Damage. Meas. J. Int. Meas. Confed..

[14] Feng B., Ribeiro A.L., Rocha T.J., Ramos H.G. (2018). Comparison of Inspecting Non-Ferromagnetic and Ferromagnetic Metals Using Velocity Induced Eddy Current Probe. Sensors.

[15] Sun L., Wu X., Ouyang Q., Wang J. (2023). A Novel Broken Wire Evaluation Method for Bridge Cable Magnetic Flux Leakage Testing under Lift-off Uncertainty. J. Magn. Magn. Mater..

[16] Sanchis R., Cardona S., Martinez J. (2018). Determination of the Vertical Vibration of a Ballasted Railway Track to Be Used in the Experimental Detection of Wheel Flats in Metropolitan Railways. J. Vib. Acoust..

[17] Pan S., Zhang D., Zhang E. Analysis of the Eccentric Problem of Wire Rope Magnetic Flux Leakage Testing. Proceedings of the 2019 IEEE 3rd Information Technology, Networking, Electronic and Automation Control Conference (ITNEC).

[18] Zhang J., Peng F., Chen J. (2020). Quantitative Detection of Wire Rope Based on Three-Dimensional Magnetic Flux Leakage Color Imaging Technology. IEEE Access.

[19] Kim J.W., Park S. (2018). Magnetic Flux Leakage Sensing and Artificial Neural Network Pattern Recognition-Based Automated Damage Detection and Quantification for Wire Rope Non-Destructive Evaluation. Sensors.

[20] Kaur A., Gupta A., Aggarwal H., Arora K., Garg N., Sharma M., Sharma S., Aggarwal N., Sapra G., Goswamy J.K. (2018). Non-Destructive Evaluation and Development of a New Wire Rope Tester Using Parallely Magnetized NdFeB Magnet Segments. J. Nondestruct. Eval..

[21] Yan X., Zhang D., Pan S., Zhang E., Gao W. (2017). Online Nondestructive Testing for Fine Steel Wire Rope in Electromagnetic Interference Environment. NDT E Int..

[22] Sun Y., Liu S., He L., Kang Y. (2017). A New Detection Sensor for Wire Rope Based on Open Magnetization Method. Mater. Eval..

[23] Yan X., Zhang D., Zhao F. (2017). Improve the Signal to Noise Ratio and Installation Convenience of the Inductive Coil for Wire Rope Nondestructive Testing. NDT E Int..

[24] Wang H., Tian J., Li X., Lv X. (2020). Inspection of Mine Wire Rope Using Magnetic Aggregation Bridge Based on Magnetic Resistance Sensor Array. IEEE Trans. Instrum. Meas..

[25] Zhang D., Zhang E., Pan S. (2020). Fractal Dimension Algorithm for MFL of Corrosion Damage in Wire Rope. Mater. Eval..

[26] Zhang J., Liu B., Zhang Z. (2022). Research on Quantitative Detection of Wire Rope Damage Based on Weak Magnetic Excitation. Russ. J. Nondestruct. Test..

[27] Ye Y., Ji K., Wang P. (2023). Influences of Magnetization Direction on the Flux Leakage Field of Weld Defects. Coatings.

[28] Coramik M., Citak H., Ege Y., Bicakci S., Gunes H. (2023). Determining the Effect of Velocity on Sensor Selection and Position in Non-Destructive Testing with Magnetic Flux Leakage Method: A Pipe Inspection Gauge Design Study with ANSYS Maxwell. IEEE Trans. Instrum. Meas..

[29] Zhou J., Tian J., Wang H., Li Y., Wu M. (2018). Numerical Simulation of Magnetic Excitation Based on a Permanent Magnet Co-Directional Array Sensor. Insight Non-Destr. Test. Cond. Monit..

[30] Wang H., Tian J., Meng G. (2017). A Sensor Model for Defect Detection in Mine Hoisting Wire Ropes Based on Magnetic Focusing. Insight Non-Destr. Test. Cond. Monit..

[31] Feng B., WU J., Qiu G., Kang Y. (2021). Development of high-speed magnetic flux leakage testing method. Nondestruct. Testing..

[32] Mazurek P. (2023). A Comprehensive Review of Steel Wire Rope Degradation Mechanisms and Recent Damage Detection Methods. Sustainability.

[33] Ji K., Wang P., Jia Y., Ye Y., Ding S. (2021). Adaptive Filtering Method of MFL Signal on Rail Top Surface Defect Detection. IEEE Access.

[34] Liu M., Zhang C., Jiang X., Sun Y., Jiang X., Li R., He L. (2022). The Research of 30 Mm Detecting Distance of Testing Device for Wire Rope Based on Open Magnetizer. Appl. Sci..

[35] Tian J., Wang H. (2015). Research on Magnetic Excitation Model of Magnetic Flux Leakage for Coal Mine Hoisting Wire Rope. Adv. Mech. Eng..

